# The Evolution of the Appendectomy: From Open to Laparoscopic to Single Incision

**DOI:** 10.6064/2012/895469

**Published:** 2012-05-27

**Authors:** Noah J. Switzer, Richdeep S. Gill, Shahzeer Karmali

**Affiliations:** ^1^Faculty of Medicine and Dentistry, University of Alberta, Edmonton, AB, Canada T6G 2R3; ^2^Department of Surgery, University of Alberta, Edmonton, AB, Canada T6G 2B7; ^3^Center for the Advancement of Minimally Invasive Surgery (CAMIS), Royal Alexandra Hospital, Edmonton, AB, Canada T5H 3V9

## Abstract

Beginning with its initial description by Fitz in the 19th century, acute appendicitis has been a significant long-standing medical challenge; today it remains the most common gastrointestinal emergency in adults. Already in 1894, McBurney advocated for the surgical removal of the inflamed appendix and is credited with the initial description of an Open Appendectomy (OA). With the introduction of minimally invasive surgery, this classic approach evolved into a procedure with multiple, smaller incisions; a technique termed Laparoscopic Appendectomy (LA). There is much literature describing the advantages of this newer approach. To name a few, patients have significantly less wound infections, reduced pain, and a reduction in ileus compared with the OA. In the past few years, Single Incision Laparoscopic Appendectomy (SILA) has gained popularity as the next major evolutionary advancement in the removal of the appendix. Described as a pioneer in the era of “scarless surgery,” it involves only one transumbilical incision. Patients are postulated to have reduced post-operative complications such as infection, hernias, and hematomas, as well as a quicker recovery time and less post-operative pain scores, in comparison to its predecessors. In this review, we explore the advancement of the appendectomy from open to laparoscopic to single incision.

## 1. Introduction

Acute appendicitis is one of the most common clinical presentations that requires emergent surgery, with a lifetime incidence of about 8% [1]. Since its first description by Fitz in 1886, much has been documented about the inflamed vermiform appendix and the need for prompt intervention to prevent the morbid consequences of perforation [2]. In the 1880s, Billroth was credited with pioneering the field of abdominal surgical intervention in Vienna, opening the door for procedures to resect diseased appendices [3]. McBurney's muscle splitting incision standardized this approach to an appendectomy upon its publication in 1894. Since then, mortality associated with acute appendicitis has been reduced to nearly 0.1% due to further improvements in medical and surgical management [4]. Surgical operations have evolved over the decades from open appendectomies to increasingly minimally invasive procedures. However, there is still ongoing discussion as to the most efficacious surgical intervention.

## 2. Discussion

### 2.1. Open Appendectomy

McBurney is credited with consolidating the surgical technique of the open appendectomy (OA) in 1894, an approach that has not significantly changed in the last 120 years [5]. 

Briefly, this conventional approach involves making an approximately 5 cm incision at the lateral border of the right rectus muscle at the midpoint between the umbilicus and right anterior-superior iliac spine [6]. Electrocautery and blunt dissection are used to separate the fascia and muscle layers, and the peritoneum is opened. The cecum then can be visualized and mobilized to reveal the appendix. The appendix and cecum are then brought out of the peritoneal cavity, the mesoappendix is ligated, and the appendiceal base is divided to leave a stump [6]. 

In 1983 with the advent of the first described minimally invasive laparoscopic appendectomy (LA) by Semm, medicine slowly shifted away from the OA. LA has become the standard of practice in uncomplicated appendectomies in most minimally invasive institutions. A US survey in 2005 showed an increased LA rate to approximately 58% of total appendectomies [7]. While LA encompasses the majority of appendectomies, the high amount of OAs still performed is quite surprising. A German study in 2009 reported nearly half of all appendectomies were open (46%) and questioned whether LA is really the standard of practice in German hospitals [8]. The surprisingly high usage of OA seen today can likely be equated to the fact that the conventional open approach has yet to be shown to be inferior to LA, as it provides reliable clinical results in a more affordable way compared to LA [9]. 

It is evident that there is still debate to the continued utility of OA. OA is widely considered the gold standard in complicated appendicitis (gangrenous and perforated appendices) due to decreased intra-abdominal infectious complications in the postoperative period [10]. It is also used as an intraoperative backup plan for LA in cases where there is severe appendiceal inflammation (the main reason for conversion to open) or if there are significant adhesions from a previous surgery—all making safe laparoscopic dissection of the appendix nearly impossible [7]. The rate of conversion from LA to OA is 8.6%, but this number is slowly decreasing as surgeons gain more experience with LA [7]. In pediatric patients under the age of 5, where the abdomen is too small for the basic physical requirement of LA, and in pregnancy, due to the risk to the fetus from LA, laparotomy is also still preferred over laparoscopy [8]. 

### 2.2. Laparoscopic Appendectomy

Briefly, the laparoscopic approach usually involves placing three ports—a 10 mm camera port at the umbilicus and 5 mm ports in the right iliac fossa and the right hypochondriac quadrant. The cecum and appendix are visualized using the camera and manipulated using a Babcock clamp, the mesoappendix is divided with an endoscopic stapler or harmonic scalpel, and the base of the appendix is ligated with either a endoscopic stapler or Endoloop. The appendix is then brought out of the peritoneal cavity using an Endobag [6]. 

Studies have shown significant advantages of this LA approach [11, 12]. Patients undergoing LA experience a reduction in wound infections, require less interoperative and postoperative pain medication, stay less time in hospital, have quickened return of normal bowel function, and improved cosmetic outcome, avoiding a large laparotomy scar. To quantify this, in a well-known meta-analysis comparing LA to OA by Sauerland et al., LA patients stayed in hospital 1.1 fewer days, returned to work 5 days earlier, experienced a reduction of pain by 8 mm on a 100 mm visual analogue scale, and experienced approximately 1/2 the number of wound infections [11]. 

Another new area of potential benefit of laparoscopy is its ability to be diagnostic, especially with reference to gynecological conditions [11, 13]. A study looking at unnecessary appendectomies in women found that in situations where a healthy-looking appendix was found and a gynecological diagnosis existed, OA had a 7-fold increased risk of removing the appendix while only making the gynecological diagnosis in 17% of its patients, versus 73% with LA patients [14]. 

In general, what to do with an uninflamed appendix, with no other clear diagnosis, is still an area of controversy. In an Italian Consensus Conference, 60% of surgeons felt that the best practice is to remove normal-looking appendices [15]. Phillips et al. found that 1/3 of all “normal looking appendices” will actually be inflamed when examined histologically [16]. However, in this study, all 18 patients, whose appendices were left in situ, did not require readmission for appendicitis by 6 months after their surgery [16]. Recently, M. N. Andresson and R. E. Andersson published a study alerting surgeons to the potential lethal consequences of performing “negative appendectomy,” stating that it was associated with an increased mortality, almost paralleling that of a perforated appendicitis [17]. Whether this changes the opinions and practices of the majority of surgeons still remains to be seen.

### 2.3. Special Populations

Four special populations, in particular, have potentially benefited from laparoscopic intervention—women, the morbidly obese, pediatrics, and geriatrics. 

The diagnostic ability of LA, as stated earlier, is especially important and useful in women, as many gynecological conditions can mimic the symptoms of appendicitis. In addition, LA is now being used as a viable option in pregnant woman. 75% of experts polled considered LA in pregnancy to be a contraindication [15]. This can be attributed to a study by McGory et al. [18] reporting that fetal loss rate was considerably higher in LA compared to OA (7% versus 3%); however recent studies have shown that LA is a safe and effective procedure in pregnancy [18–20]. 

Surgery on the morbidly obese is more challenging compared to the rest of the population. Varela et al. reported that OA was preferred more often to LA (53% to 47%) due to this inherited difficulty. However, in their retrospective review of 1,943 morbidly obese patients, LA was associated with a shorter length of hospitalization, lower cost, and lower postoperative complications (particularly wound infections) [21]. Another study by Woodman et al. stated a 50% reduction in morbidity with LA [22]. As the largest study comparing the two interventions to date, Varela et al. concluded their study by recommending LA over OA as the treatment of choice for all clinical presentations of appendicitis (perforated or not, high risk patient or not) unless specifically contraindicated [21]. 

Lastly, appendectomy is the most common surgical emergency in the pediatric population [23, 24]. The literature is now reporting that children after LA are able to return to their normal activities quicker compared to OA and have less postoperative pain score and complications [24, 25]. Therefore, it seems to be a more successful procedure for children, as long as their abdomens can physically support laparoscopic procedures (over the age of 5 years old). At the other extreme of the population, the literature has shown that the elderly have a mortality reduction of nearly 1% after LA compared with OA and a lower overall reduction in complication rate (15% versus 23%) [26]. 

While there appears to be many advantages of LA, there are also significant disadvantages. After LA, patients are 3 times more likely to have an intra-abdominal abscess. Bonnani et al. found that almost 50% of patients with complicated appendicitis, treated by LA had to be readmitted for infectious complications [10]. As well, operating time for LA is about 10 minutes longer than with a laparotomy. However, recent studies have shown that as experience with laparotomy increases, operating time has decreased, culminating in LA being only approximately 5 minutes longer [12]. LA is also a more expensive surgery than its conventional counterpart. But some studies have argued that while there is increased operating cost with LA, it is balanced by the patients' early return to work, decreasing cost at a societal level.

Of note, Sauerland et al. mention several possible limitations of randomized controlled trials in their ability to adequately compare LA with OA [11]. The authors state that since LA has the ability to be diagnostic, there are situations in which an appendectomy was never carried out, biasing the results towards faster operating times. As well, they argue that the stated reduction in hospital stay and pain scores seen in LA, while statistically significant, perhaps are not clinically significant [11].

It is safe to say that many important patient and institution factors need to be taken into consideration for the decision algorithm of which type of intervention to perform. These include but are not limited to what equipment is available, the level of experience of the operator, the severity of the appendicitis and likelihood of post-operative complications.

### 2.4. Single Incision Laparoscopic Appendectomy

In 1992, Pelosi first described a single-puncture laparoscopic appendectomy in 25 patients [27]. However, it was not until the last few years that this new minimally invasive technique called the single incision laparoscopic appendectomy (SILA) really caught on. It has been proposed as the next major breakthrough in the appendectomy evolution. 

The surgical technique for SILA is not yet standardized, with great institutional procedural variation. Briefly, SILA involves a 2-3 cm incision usually transumbilically, but can also be made at McBurneys point, and inserting the laparoscope and surgical laparoscopic instruments via a 10 mm and multiple 5 mm ports [28, 29]. In addition, a needlescopic instrument can be placed percutaneously in the right iliac fossa for assistance in supporting the appendix [28]. Either rigid conventional laparoscopic instruments can be used or special bendable instruments [28, 30, 31]. The mesoappendix is then divided with the appendiceal artery cauterized and the base of the appendix ligated with an Endloop. The appendix is then removed through the 10 mm port. 

The biggest advantage of this new type of technique is in its cosmetic outcome; it is being referred to as “scarless surgery.” In addition, the expectation is that a reduction in the number of surgical incisions will correlate to both a decline in incisional complications like infections, hernias, and hematomas, as well as a decrease in adhesion formation and improvement in patient convalescence [32, 33]. 

To date, there is no published randomized controlled trial (RCT) comparing SILA with LA or OA [34]. Therefore, any comparisons between LA and SILA must be done retrospectively. There was, however, an RCT of 40 patients comparing the standard laparoscopic cholecystectomy versus single incision laparoscopic cholecystectomy, looking at pain scores after surgery. It was found that single incision patients reported significantly less post-operative abdominal pain [35]. Greaves and Nicholson compared SILA and OA and found that patients had similar durations of stay and pain scores after SILA, but that single incision procedures had increased operating time [32]. However, in contrast to what was expected, Chow et al. reported that although SILA is a more technically challenging procedure, it took significantly less operating time (60 minutes) when compared to LA (70 minutes) [36]. This paradoxical finding was largely felt to be attributed to the staff surgeon usually performing the appendectomies over the learner, as most residents are not yet comfortable with elements of SILA—the limitation in instrument triangulation, the increased susceptibility of instrument collisions, and a reduction in the visual field [33, 36]. There is already new literature that is orientated to improving upon these limitations. One of the proposed mechanisms is called the magnetic anchoring guidance system, which involves a magnet and camera apparatus that can move unrestricted intra-abdominally, not relying on a fixed camera port thereby limiting instrument collision and restoring some of the natural triangulation seen with LA [33]. 

As stated earlier, SILA can be performed using specialized equipment or with conventional ones. The specialized equipment can put a strain on hospital resources, as they are more expensive, making SILA a more impractical procedure [37]. On the other hand, a cost-benefit analysis of the operation, using conventional laparoscopic equipment, found SILA to be quite similar to LA in the total disposable instrument cost, both around $800 USD [36]. 

There is much heterogeneity in the studies comparing LA with SILA, as well as with the surgical technique itself. It appears that these two techniques have similar outcomes, but an RCT is required to be fully comfortable with comparison. Interestingly, some surgeons have recently described using LA and SILA in sequence. An operation can be started using the single incision approach and depending on how complicated the appendectomy appears; ports may be added to convert the procedure to LA [38]. 

### 2.5. Future Techniques

SILA is only a stepping stone to what lies ahead for minimally invasive surgery, possibly to a technique called natural orifice translumenal endoscopic surgery (NOTES), which involves no external scarring [36]. However, while minimally invasive surgery continues to make great strides, it is important to recognize that perhaps the best surgical intervention for appendicitis is no surgery at all. There is growing evidence in the literature promoting conservative treatment of appendicitis, strictly with antibiotics. Varadhan et al. found that antibiotics are a safe initial management plan for acute appendicitis, with a 63% success rate and a significant risk reduction in complications compared with appendectomy [39]. 

## 3. Conclusion

Surgical advancement in the management of appendicitis has evolved dramatically in the last 120 years, from McBurney's simple large incision, to minimally invasive LA, to barely noticeable incisions after SILA. Depending on the clinical situation and the experience of the surgeon, each of the three techniques (OA, LA, and SILA) can be effective. Minimally invasive surgery will continue to push the limits.

## References

[1] Addiss DG, Shaffer N, Fowler BS, Tauxe RV (1990). The epidemiology of appendicitis and appendectomy in the United States. *American Journal of Epidemiology*.

[2] Fitz RH (1886). Perforating inflammation of the vermiform appendix with special reference to its early diagnosis and treatment. *Transactions of the Association of American Physicians*.

[3] Buklijas T (2007). Surgery and national identity in late nineteenth-century Vienna. *Studies in History and Philosophy of Science Part C*.

[4] Worni M, Ostbye T, Gandhi M (2012). Laparoscopic appendectomy outcomes on the weekend and during the week are no different: a national study of 151,774 patients. *World Journal of Surgery*.

[5] McBurney C (1894). The incision made in the abdominal wall in cases of appendicitis, with a description of a new method of operating. *Annals of Surgery*.

[6] Townsend CM, Ever B (2010). *Atlas of General Surgical Techniques*.

[7] Sakpal SV, Bindra SS, Chamberlain RS (2011). Laparoscopic cholecystectomy conversion rates two decades later: an analysis of surgeon and patient-specific factors resulting in open conversion. *Journal of the Society of Laparoendoscopic Surgeons*.

[8] Reißfelder C, Cafferty BM, von Frankenberg M (2009). Open appendectomy. When do we still need it?. *Chirurg*.

[9] Cariati A, Brignole E, Tonelli E (2001). Laparoscopic or open appendectomy. Critical review of the literature and personal experience. *Giornale di Chirurgia*.

[10] Bonanni F, Reed J, Hartzell G (1994). Laparoscopic versus conventional appendectomy. *Journal of the American College of Surgeons*.

[11] Sauerland S, Lefering R, Neugebauer EA (2002). Laparoscopic versus open surgery for suspected appendicitis. *Cochrane Database of Systematic Reviews*.

[12] Bennett J, Boddy A, Rhodes M (2007). Choice of approach for appendicectomy: A meta-analysis of open versus laparoscopic appendicectomy. *Surgical Laparoscopy, Endoscopy and Percutaneous Techniques*.

[13] Horstmann R, Tiwisina C, Classen C, Palmes D, Gillessen A (2005). Laparoscopic versus open appendectomy: Which factors influence the decision between the surgical techniques?. *Zentralblatt fur Chirurgie*.

[14] Larsson PG, Henriksson G, Olsson M (2001). Laparoscopy reduces unnecessary appendicectomies and improves diagnosis in fertile women: a randomized study. *Surgical Endoscopy*.

[15] Vettoretto N, Gobbi S, Corradi A (2011). Consensus conference on laparoscopic appendectomy: Development of guidelines. *Colorectal Disease*.

[16] Phillips AW, Jones AE, Sargen K (2009). Should the macroscopically normal appendix be removed during laparoscopy for acute right iliac fossa pain when no other explanatory pathology is found?. *Surgical Laparoscopy, Endoscopy and Percutaneous Techniques*.

[17] Andersson MN, Andersson RE (2011). Causes of short-term mortality after appendectomy: a population-based case-controlled study. *Annals of Surgery*.

[18] McGory ML, Zingmond DS, Tillou A, Hiatt JR, Ko CY, Cryer HM (2007). Negative appendectomy in pregnant women is associated with a substantial
risk of fetal loss. *Journal of the American College of Surgeons*.

[19] Jeong JS, Ryu DH, Yun HY, Jeong EH, Choi JW, Jang LC (2011). Laparoscopic appendectomy is a safe and beneficial procedure in pregnant women. *Surgical Laparoscopy, Endoscopy and Percutaneous Techniques*.

[20] Eom JM, Hong JH, Jeon SW (2012). Safety and clinical efficacy of laparoscopic appendectomy for pregnant women with acute appendicitis. *Annals Academy of Medicine Singapore*.

[21] Varela JE, Hinojosa MW, Nguyen NT (2008). Laparoscopy should be the approach of choice for acute appendicitis in the morbidly obese. *American Journal of Surgery*.

[22] Woodham BL, Cox MR, Eslick GD Evidence to support the use of laparoscopic over open appendicectomy for obese individuals: a meta-analysis.

[23] Schmelzer TM, Rana AR, Walters KC, Norton HJ, Bambini DA, Heniford BT (2007). Improved outcomes for laparoscopic appendectomy compared with open appendectomy in the pediatric population. *Journal of Laparoendoscopic and Advanced Surgical Techniques*.

[24] Aziz O, Athanasiou T, Tekkis PP (2006). Laparoscopic versus open appendectomy in children: a meta-analysis. *Annals of Surgery*.

[25] Lintula H, Kokki H, Vanamo K, Valtonen H, Mattila M, Eskelinen M (2004). The costs and effects of laparoscopic appendectomy in children. *Archives of Pediatrics and Adolescent Medicine*.

[26] Masoomi H, Mills S, Dolich MO (2012). Does laparoscopic appendectomy impart an advantage over open appendectomy in elderly patients. *World Journal of Surgery*.

[27] Pelosi MA, Pelosi MA (1992). Laparoscopic appendectomy using a single umbilical puncture (minilaparoscopy). *Journal of Reproductive Medicine for the Obstetrician and Gynecologist*.

[28] Bhatia P, Sabharwal V, Kalhan S, John S, Deed J, Khetan M (2011). Single-incision multi-port laparoscopic appendectomy: how i do it ?. *Journal of Minimal Access Surgery*.

[29] Chen D, Shi H, Dong H, Liu K, Ding K (2011). Gasless single-incision laparoscopic appendectomy. *Surgical Endoscopy and Other Interventional Techniques*.

[30] Chiu CG, Nguyen NH, Bloom SW (2011). Single-incision laparoscopic appendectomy using conventional instruments: an initial experience using a novel technique. *Surgical Endoscopy and Other Interventional Techniques*.

[31] Brunner W, Schirnhofer J, Waldstein-Wartenberg N, Frass R, Pimpl K, Weiss HG (2009). New: single-incision transumbilical laparoscopic surgery. *European Surgery*.

[32] Greaves N, Nicholson J (2011). Single incision laparoscopic surgery in general surgery: a review. *Annals of The Royal College of Surgeons of England*.

[33] Cadeddu J, Fernandez R, Desai M (2009). Novel magnetically guided intra-abdominal camera to facilitate laparoendoscopic single-site surgery: initial human experience. *Surgical Endoscopy and Other Interventional Techniques*.

[34] Rehman H, Rao AM, Ahmed I (2011). Single incision versus conventional multi-incision appendicectomy for suspected appendicitis. *Cochrane Database of Systematic Reviews*.

[35] Tsimoyiannis EC, Tsimogiannis KE, Pappas-Gogos G (2010). Different pain scores in single transumbilical incision laparoscopic cholecystectomy versus classic laparoscopic cholecystectomy: a randomized controlled trial. *Surgical Endoscopy and Other Interventional Techniques*.

[36] Chow A, Purkayastha S, Nehme J, Darzi LA, Paraskeva P (2010). Single incision laparoscopic surgery for appendicectomy: a retrospective comparative analysis. *Surgical Endoscopy and Other Interventional Techniques*.

[37] Tam YH, Lee KH, Sihoe JDY, Chan KW, Cheung ST, Pang KKY (2010). Initial experience in children using conventional laparoscopic instruments in single-incision laparoscopic surgery. *Journal of Pediatric Surgery*.

[38] Hong TH, Kim HL, Lee YS (2009). Transumbilical single-port laparoscopic appendectomy (TUSPLA): scarless intracorporeal appendectomy. *Journal of Laparoendoscopic and Advanced Surgical Techniques*.

[39] Varadhan KK, Neal KR, Lobo DN (2012). Safety and efficacy of antibiotics compared with appendicectomy for treatment of uncomplicated acute appendicitis: meta-analysis of randomised controlled trials. *BMJ*.

